# Endostar plus pembrolizumab combined with a platinum-based dual chemotherapy regime for advanced pulmonary large-cell neuroendocrine carcinoma as a first-line treatment: A case report

**DOI:** 10.1515/biol-2022-0062

**Published:** 2022-06-15

**Authors:** Sijia Zhang, Yu Xiao, Leichong Chen, Zhenyu Li, Yan Zong, Kuikui Zhu, Rui Meng

**Affiliations:** Department of Oncology, Cancer Center, Union Hospital, Tongji Medical College, Huazhong University of Science and Technology, 1277 Jiefang Avenue, Wuhan, 430022, People’s Republic of China

**Keywords:** pulmonary large-cell neuroendocrine carcinoma, immunotherapy, anti-angiogenesis, chemotherapy, combination therapy

## Abstract

Pulmonary large-cell neuroendocrine carcinoma (LCNEC) is a rare and highly aggressive cancer with a very poor prognosis. The proper treatment decision and possible prognosis outcome for advanced LCNEC is always an enormous challenge due to its scarcity. Here, we presented a 59-year-old male patient with advanced LCNEC with a non-neuroendocrine immunophenotype who received endostar plus pembrolizumab combined with a platinum-based dual chemotherapy regime as a first-line treatment. At present, the patient’s condition is well controlled by medication only and has a progression-free survival of more than 2 years. Adverse effects recorded for this patient during treatment courses include nausea, vomiting, II–III quality bone marrow toxicity, and PD-1 blockage-related hypothyroidism. This case report discussed the feasibility of immunotherapy, anti-angiogenesis agents, and chemotherapy as a first-line therapy in advanced LCNEC.

## 1 Introduction

Pulmonary large-cell neuroendocrine carcinoma (LCENC) is a high-grade neuroendocrine (NE) carcinoma, accounting for 1.7–3.0% of all diagnosed lung cancer [1]. Liver (47%), bone (32%), and brain (23%) metastases were found at initial diagnosis in more than half of LCNEC patients, with a median overall survival (mOS) of 4.0 months [95% confidence interval (CI) 3.5–4.6] [1]. Up to date, the optimal treatment strategy for LCNEC is rarely known. Due to the similarity to small-cell lung carcinoma (SCLC) in clinical characteristics, common LCENC is treated with SCLC-like platinum-based doublet chemotherapeutic regimens, such as cisplatin–etoposide or cisplatin–irinotecan [2]. However, poor control rates and low progression-free survival (PFS)/overall survival (OS) are still important limitations.

Immunotherapy, PD-1/PD-L1 blockade, and anti-angiogenesis agents have antitumor activity. Synergic therapy of the above drugs with cytotoxic agents has been confirmed to prolong the survival time of patients with NSCLC and/or SCLC as first-line therapy. Currently, the efficiency of synergic therapy is still unclear in metastasis LCNEC. Here, we reported a case of advanced LCNEC that responded well to irinotecan–nedaplatin chemotherapy combined with immunotherapy and antiangiogenic as the first-line therapy.

## 2 Case presentation

**Figure 1:**
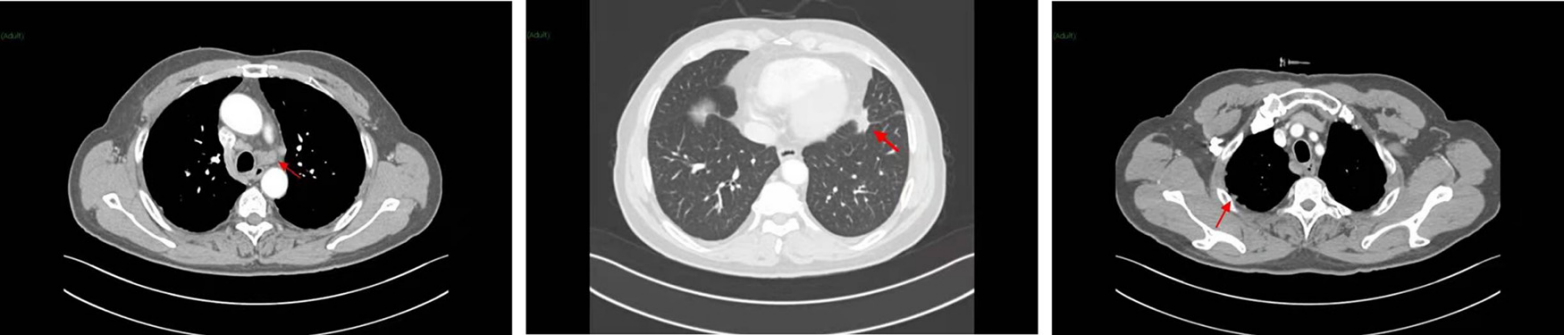
Chest CT scan revealed multiple solid small nodules were scattered in both lungs, multiple nodules in bilateral pleura, multiple enlarged lymph nodes in mediastinum and bilateral hilum. A patchy soft tissue density shadow with a cross-sectional area of about 1.6 cm × 0.9 cm was seen near the heart margin of the lingual segment of the left lung, which was significantly enhanced by enhanced scan.

**Figure 2:**
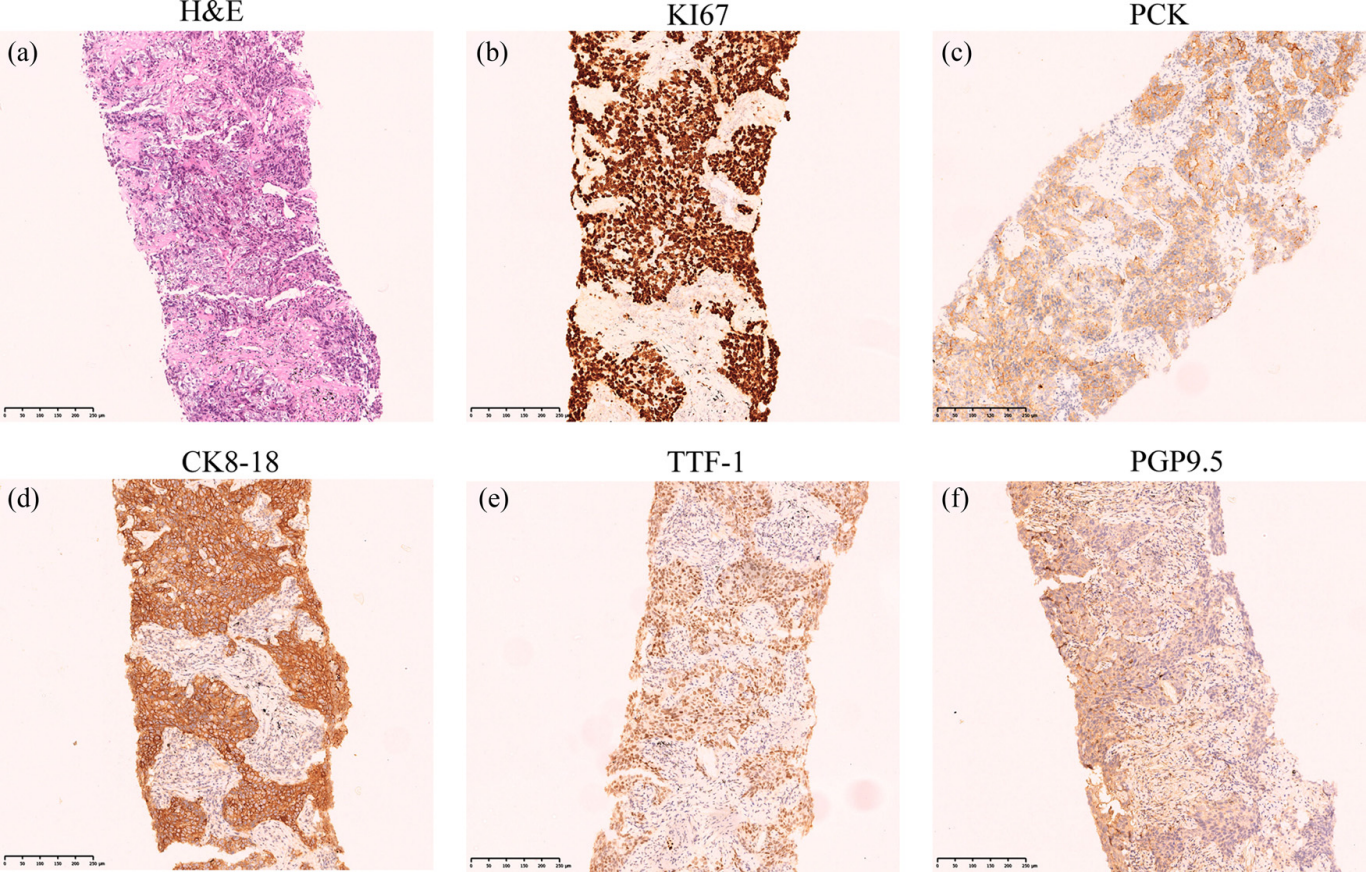
Morphologic and IHC features. (a) Pathological examination of puncture biopsy of the left lung by hematoxylin and eosin (H&E) (100× magnification), scale bar 250 μm, (b–f) IHC positive staining for KI67 (∼90% of the cells stained), PCK (punctate beside the nucleus), CK8-18, TIF-1 and PGP 9.5, (100× magnification), scale bar 250 μm.

In May 2018, a 59-year-old male patient was diagnosed at a local hospital with left lung space and left pleural effusion due to sudden left chest pain. He was admitted to our hospital (Union Hospital of Tongji Medical College, Wuhan, China) the next month. The patient complained of hypertension and long history of smoking with a smoking index of over 400. Admission physical examination revealed no positive signs and the Eastern Cooperative Oncology Group (ECOG) performance status of the patient was 1. A positron emission tomography–computed tomography (CT) scan showed multiple nodules in the lingual segment of the left upper lobe, with a maximum of 2.1 cm × 2.2 cm, which was closely related to the pleura. Part of them had abnormally increased metabolism. Bilateral pleural and interlobular fissures were unevenly thickened, and multiple nodules were concentrated on the left side with increased metabolic dispersion. Multiple lymph nodes enlargement occurred in the mediastinum (areas 3R, 4R/L, 5, 7) and hilar of both lungs with abnormal metabolism (Figure 1). Malignant tumor lesions are mostly considered according to those mentioned above. The primary left lung with bilateral pleura, interlobular fissure, mediastinum, and bilateral hilar lymph node metastasis was likely to be left pleural effusion; there were no signs of tumor metastasis in the rest of the exploration site. On June 13, 2018, a CT-guided biopsy of the left lung was performed. Histopathology showed a non-small-cell carcinoma with NE morphology (shown in Figure 2a), which was suspected to be LCENC, but the NE marker was negative. Immunohistochemical (IHC) results are summarized in Table 1, and all the positive staining is presented in Figure 2b–f. PD-L1 expression status was not tested due to the deficiency of the puncture biopsy. Brain magnetic resonance imaging (MRI) excluded brain metastases. Next-generation sequencing (NGS) analysis using 556 cancer-related gene panels revealed a total of 6 non-synonymous somatic mutations (4 missenses, 2 introns). Based on the above results, this patient was diagnosed with c-stage IVA, cT2N3M1b (pleura) (AJCC8th TNM classification).

**Table 1:** Results of IHC staining

Immunohistochemistry	Status
**Puncture biopsy of the left lung (tumor cells)**	
PCK (paranuclear punctate+), CK8/18, TTF-1, PGP9.5, Ki67 (Li90%)	Positive
CD117, SOX10, Syn, CgA, CD56, CK7, NapsinA, P40	Negative
**Pericardial effusion sediment (dyskaryotic cells)**	
Claudin-4, PCK	Positive
CK8/18, Calretinin, CK5/6, CD68, TTF-1, PGP9.5	Negative

**Figure 3:**
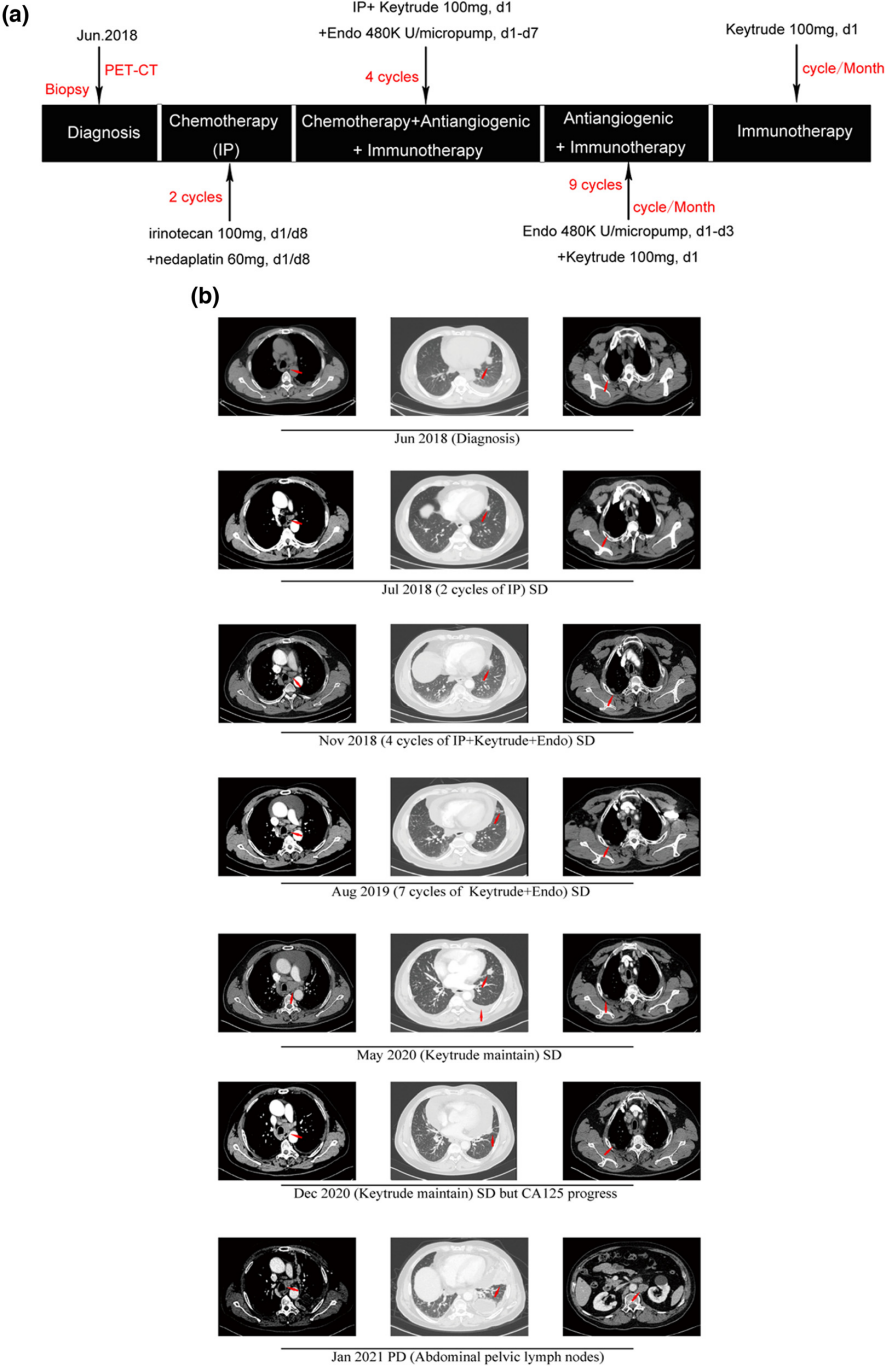
Treatment process and therapeutic effect. (a) Schematic diagram of the patient’s clinical treatment process. (b) Chest CT (computed tomography) scanning. PD, progressive disease.

**Figure 4:**
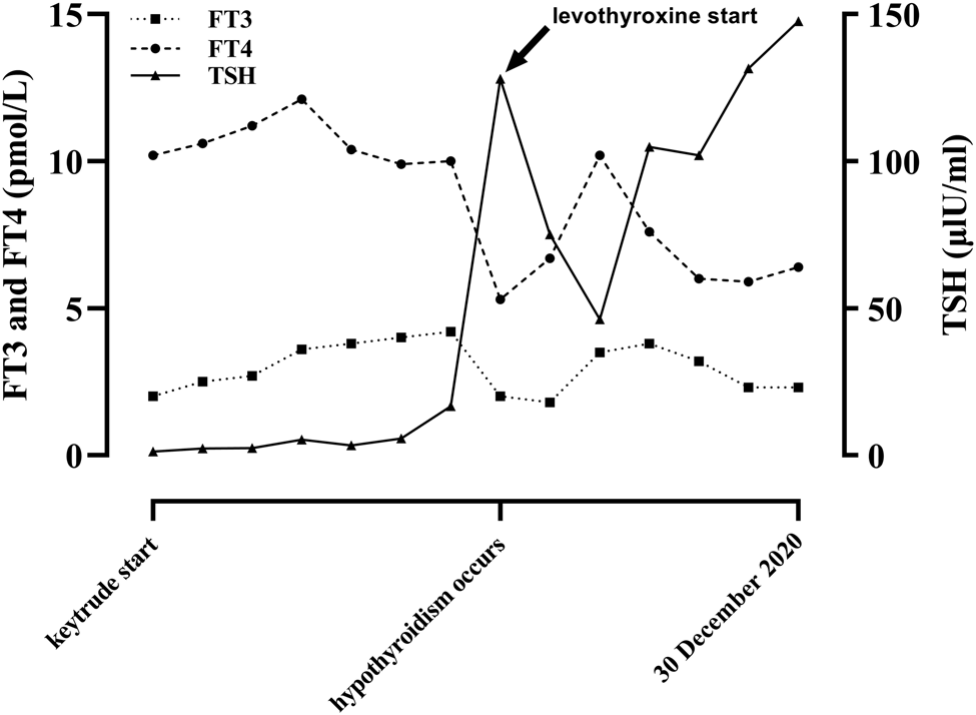
Time course of thyroid function of the patient during treatment with pembrolizumab.

A standard platinum-based doublet chemotherapy regime (nedaplatin 60 mg and irinotecan 100 mg) was utilized for two treatment cycles. Post-treatment CT showed stable disease (SD). To make the persistent control of the patient’s condition, standard chemotherapy was used in combination with both 100 mg pembrolizumab once per cycle and continual 48KU/micropump endostar 1 week per cycle. The platinum-based regime was abandoned after four cycles. One hundred milligrams of pembrolizumab once per cycle and continual 48KU/micropump endostar 1 week per cycle continued nine cycles. During the whole treatment, CT still showed SD, which is shown in Figure 3a and b. Occasionally nausea and discomfort that occurred in a patient during the chemotherapy could be relieved by himself. Hypothyroidism associated with PD-1 blockade was diagnosed in a patient after 11 cycles of using pembrolizumab and was rapidly relieved by oral administration of thyroxine tablets. Serum levels of FT3, FT4, and TSH during this period are shown in Figure 4 and Table A1.

**Figure 5:**
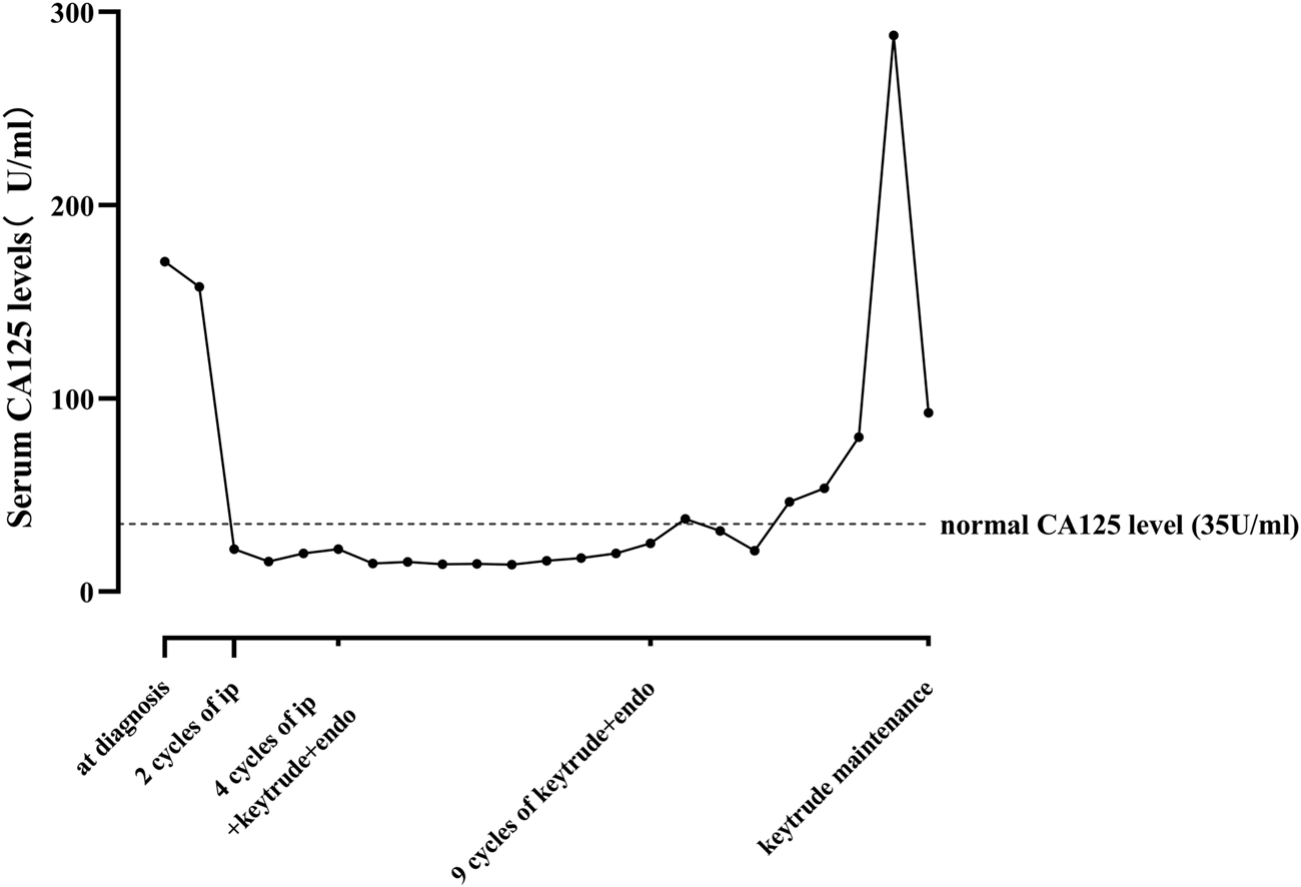
The patient’s CA125 dropped to normal after treatment. Then slowly elevated from 46.4 to 79.9 ng/mL in the SD period. Up to Dec 2020, CA125 rapidly jumped to 287.8 ng/mL.


The maintenance of the treatment plan was discontinued from January 2020 to May 2020 due to the unexpected COVID-19. On May 27, 2020, the patient returned to our hospital and was found with a large amount of pericardial effusion. Fortunately, pericardial puncture and drainage and pericardial perfusion therapy maintained good control of effusion. Subsequent pathological examination of pericardial drainage fluid sediment showed a very few dyskaryotic cells. Immunohistochemistry was then performed and the results were as follows: paranuclear punctate (PCK) (+), CK8/18 (+), TTF-1 (+), PGP9.5 (+), Ki67 (+), CD117 (−), SOX10 (−), Syn (−), CgA (−), CD56 (−), CK7 (−), NapsinA (−), and P40 (−) in the tumor cells and Claudin-4 (+), PCK (+), CK8/18 (−), Calretinin (−), CK5/6 (−), CD68 (−), TTF-1 (−), and PGP9.5 (−) (shown in Table 1). In addition, no new lesions were found in neck, chest, and abdomen enhanced CT, brain enhanced MRI, and systemic bone emission computed tomography ECT as shown in Figure 3b. However, the serum CA125 level was elevated to 46.4 ng/mL. Therefore, experts of in-hospital MDT (multi-disciplinary team) were required to reevaluate his situation, but there was no adequate evidence to support disease progression. These experts advised that the patient should continuously receive 100 mg pembrolizumab once per cycle. Thereafter, the disease remained stable under observation for approximately 6 months, during which the CA125 level slowly increased from 46.4 to 79.9 ng/mL. Up to December 2020, CA125 rapidly increased to 287.8 ng/mL as shown in Figure 5. The patient’s disease progressed after 30 months of antitumor treatment.

**Informed consent:** Informed consent has been obtained from all individuals included in this study.
**Ethical approval:** The research related to human use has been complied with all the relevant national regulations and institutional policies and in accordance with the tenets of the Declaration of Helsinki and has been approved by the authors’ institutional review board or equivalent committee.

## 3 Discussion

Pulmonary LCENC is a high-grade non-small-cell NE carcinoma classified as a variant of large-cell carcinoma in the updated WHO classification of lung tumors [3]. There are four major types of NE phenotypes in large-cell carcinoma. One of them is large-cell carcinoma with NE morphology but non-NE immunophenotype [4], which is generally similar to LCENC [5,6]. The optimal chemotherapy regimen of stage IV pLCNEC as a first-line treatment remains controversial. Because LCNEC and SCLC have similar neuroendocrine morphology and biomarker expression, it is difficult to distinguish the difference between them. Generally speaking, there is no clear standard treatment for LCNEC in clinics and some scholars recommend EP as the first choice for treatment. Our case report showed an experience of treatment of advanced pLCENC without NE phenotype as the first-line therapy.

In the first-line treatment, two prospective single-arm phase II studies for advanced LCNEC that performed a platin-based doublet regimen of SCLC like had a positive effect during the short term. One study enrolled a total of 44 patients with the treatment of cisplatin–irinotecan, and the response rate was 54.5%, with the median PFS (mPFS) and mOS of 5.9 and 15.1 months, respectively [7]. Another trial showed an objective response rate of 38% and a disease control rate of 64% among 42 patients with cisplatin and etoposide treatment. The mPFS and mOS were 5.3 and 7.7 months, respectively [8].

With the development of NGS technology, studies of the genetic environment in LCNEC have explained a series of variants that differed from normal tissue and brought great benefits in identifying therapeutic drugs. George et al. provided a very comprehensive multi-omics analysis of LCNEC and revealed *TP53* (92%), *RB1* (42%), *LKB1*(*STK11*) (30%), as well as *KEAP1* (22%) mutations [9]. Furthermore, they found that loss of heterozygosity, biallelic alterations, and larger genomic rearrangements were the main reasons for somatic alterations of RB1 and STK11/KEAP1, and these were mutually exclusive. Thus, two separate subtypes can be divided molecularly based on mutation evidence. Type I is defined by *STK11/KEAP1* alterations and has an NE phenotype with the high expression of *ASCL1* and *DLL3* but the downregulation of the *NOTCH* pathway. In contrast, type II is characterized by *RB1* alterations but predominantly non-NE phenotype, high expression of *REST* and *NOTCH*, and activation of immune cell response. Lantuejoul et al. indicated that type I can benefit from a treatment of NSCLC chemotherapy ± IC inhibitors and type II from SCLC treatment [10]. *RB1* expression by IHC staining is another significant evaluation indicator. Derks et al. showed that pLCNEC patients with *RB1* wild-type and/or expressing *RB1* have superior overall survival when treated with NSCLC-like chemotherapy compared to SCLC-like (9.6 vs 5.6 months), but with no difference in the outcome for patients with inactivated *RB1* [11]. Our patient accepted NGS analysis and revealed a total of six non-synonymous somatic mutations (four missenses, two introns). Unfortunately, none of the related gene mutations reported above were found. According to these results, we preliminarily speculated that the better response of immunotherapy might be related to the type of the detected mutations because some patients with LCNEC are not effective against immune checkpoint inhibitor drugs. Like literature reported by Han et al., this patient (staged at IIIA with pLCNEC) was treated with thoracoscopic radical surgery for upper left lung cancer and postoperative chemotherapy with a double-drug regimen holding platinum. Then, bevacizumab, paclitaxel, and the PDL1 checkpoint inhibitor nivolumab were applied, but the patient’s disease progressed rapidly. Even with positive PD-L1 expression, immunotherapy is an ineffective treatment for these patients. Researchers thought that a possible contributing factor is the timing of immunotherapy too late [12]. In addition, the tumor was effectively controlled when the patient in our study achieved nedaplatin-irinotecan agents. We speculated that the molecular phenotype of the patient might be SCLC like because of non-NE markers and high Ki-67 index (90%), but the platinum-based dual regimen did not make his tumor essentially regress. The risk of recurrence in the short term because of potential SCLC-like characteristics. Among other variant targets, *KRAS* mutations in LCENC are the most common (approximately 22–24%) and can be associated with adverse responses to chemotherapy [13,14]. Nonetheless, EGFR and BRAF mutations in LCNEC are very low, nearly 2 and 1%, respectively, which is different from NSCLC [14]. Therefore, *EGFR*-TKIs may not be satisfactory for the efficacy of LCNEC.

In immunotherapy, PD-1/PD-L1 inhibitors, which activate natural antitumor immune responses by disrupting the linkage of PD-1 to PD-1 ligand, have been observed to be effective in treating NSCLC, SCLC, and/or LCNEC. Studies by Sherman et al. [15] and Levra et al. [16] showed that single-agent immune checkpoint inhibitors (ICI) as a second line or multiple line for the treatment of patients with advanced LCNEC were associated with 33 and 60% of total ORR, respectively. Eleven percentage (2/21) of patients had a complete response (CR) in Sherman’s study. mPFS was 4.2 months and 57 weeks. In addition, synergistic regimens of ICI plus chemotherapy have not only been testified to shrink tumor diameter and achieve a major pathological response (MPR) in the period of neoadjuvant therapy [17] but also have been found to control disease progression[18] in advanced LCNEC. To date, information on the use of PD-1/PD-L1 inhibitors in first-line advanced LCNEC is very sparse. Recently, PD-L1 blockades, atezolizumab, and durvalumab in combination with platinum-based chemotherapy have sequentially been approved for the first-line treatment of extensive-stage SCLC (ES-SCLC) patients deriving from Impower130 trail and CASPIN trail, respectively. It implied that metastatic LCNEC, like ES-SCLC, could be used in synergic therapy with immunochemotherapy as the first-line therapy. Both PD-L1 expression and tumor mutation burden (TMB) are important biomarkers for response to immunotherapy in NSCLC or SCLC. In advanced LCNEC, several prior studies reported that PD-L1 expression rates were 10–20% [19,20]. The median TMB has been represented to be 9.9 muts/Mb in pLCNEC [21], compared to NSCLC (median 5.7 muts/Mb) [19], which indicated that LCNEC patients have a potential advantage in making a PD-1/PD-L1 inhibitor efficient. Zhang et al. described an LCENC patient with high TMB (25.8 muts/Mb) but negative PD-L1 who achieved a CR after nivolumab treatment when the tumor rapidly progressed after surgery and adjuvant therapy [22]. Xu et al. reported a case of a patient who was staged at cT1bN3M0 IIIB with locally advanced pulmonary LCNEC. After surgical resection and chemoradiotherapy, the patient achieved complete remission. PD-L1 (+) results were >50% in this case, so PD-L1 inhibitor durvalumab was then started to consolidate the treatment. After six courses of immune maintenance therapy, the patient developed grade 2 immune-related pneumonitis and took prednisone orally until the symptoms resolved, and then reached complete remission again [12,23]. In our case, TMB is 2.77 muts/Mb, and PD-L1 expression is undetected due to the inadequacy of biopsy. To strive for more than survival time, pembrolizumab was synergized with nedaplatin-irinotecan treatment for our patient. However, low TMB for our patient prompted the integration of other treatment manners.

Angiogenesis inhibitors directly fight against vascular endothelial growth factor (VEGF), which influences tumor growth inhibition. In the GOIRC-AIFA trial, bevacizumab was added to the first-line dual chemotherapy for ED-SCLC treatment, which showed a small, statistically significant improvement in PFS (5.7 vs 6.7 months; hazard ratio, 0.72; 95% CI, 0.54–0.97), but with no difference in OS [24]. In pLCENC, angiogenesis inhibitors are rarely experienced. A few studies reported overexpression of VEGF in pLCENC. Iyoda et al. explored potential molecular targets in pLCNEC, and they found that 13/13 (100%) tumors exhibited high VEGF expression as compared to 13/14 (92.9%) tumors of lung adenocarcinomas (ACs), suggesting a possible role for anti-VEGF therapy in the treatment of pLCNEC [25]. Recently, Muto et al. [26] reported a salvage therapy case with combined LCNEC with adenocarcinoma harboring an *EGFR* mutation (exon19, E746–A750 deletion) achieved afatinib, erlotinib, and bevacizumab treatment, and the disease remained stable for 10 months [25]. Meanwhile, Wu et al. confirmed anti-angiogenesis agent, endostar, collaborated with PD-1 blockade and had a dramatically synergic effect to suppress tumor growth in the LCC mice model through up-regulating PI3K/AKT/mTOR-mediated autophagy and angiogenesis [27]. In the first-line treatment, our patient took a synergistic method, with endostar and immunochemotherapy and reached a persistent SD over 30 months.

In conclusion, our case provides a potentially effective and safe treatment option in advanced and/or metastasis pulmonary LCNEC as the first-line therapy. The limitation of our study is that we reported only one case, which was insufficient to further explore the OS, PFS, and other indices for this malignant disease. Perhaps, the model can be appropriate in a herd of patients with IV stage pLCENC with negative NE markers, high mitotic rates, no *RB1* and/or *TP53* mutations, high TMB, or positive PD-L1 expression. However, it is difficult to draw definitive conclusions based on such limited experience. Our case report may provide a reference for treatment among our peers. At present, immunotherapy has made a breakthrough for NSCLC patients, and it is expected that more clinical studies will be conducted to explore its effectiveness in the treatment of LCNEC.

## Appendix

**Table A1:** The serum CA125, FT3, FT4, and TSH levels during the patient’s diagnosis and treatment

Number	Date	CA125		Number	Date	TSH	FT3	FT4
1	2018.6.4	170.7		1	2018.7.31	1.2882	2	10.2
2	2018.7.5	157.8		2	2018.8.26	2.2358	2.5	10.6
3	2018.7.27	21.9		3	2018.10.15	2.4252	2.7	11.2
4	2018.8.21	15.6		4	2018.11.22	5.2652	3.6	12.1
5	2018.9.18	19.8		5	2018.12.25	3.3175	3.8	10.4
6	2018.10.15	21.9		6	2019.1.29	5.7141	4	9.9
7	2018.11.22	14.6		7	2019.4.9	16.6002	4.2	10
8	2018.12.25	15.3		8	2019.8.6	128.048	2	5.3
9	2019.1.29	14.2		9	2019.9.5	75.041	1.8	6.7
10	2019.3.5	14.4		10	2019.10.12	46.2223	3.5	10.2
11	2019.4.9	14		11	2020.5.28	104.7989	3.8	7.6
12	2019.5.17	16		12	2020.7.22	101.9509	3.2	6
13	2019.6.27	17.3		13	2020.12.2	131.5156	2.3	5.9
14	2019.8.6	19.8		14	2020.12.30	147.5986	2.3	6.4
15	2019.9.5	25						
16	2019.10.12	37.5						
17	2019.11.19	31.3						
18	2019.12.24	21.2						
19	2020.5.28	46.4						
20	2020.7.22	53.4						
21	2020.12.2	79.9						
22	2020.12.30	287.8						
23	2021.1.23	92.6						
